# Influence of body size on platelet response to ticagrelor and prasugrel in patients with acute coronary syndromes

**DOI:** 10.1007/s00392-021-01976-y

**Published:** 2021-12-01

**Authors:** Gjin Ndrepepa, Stefan Holdenrieder, Isabell Bernlochner, Adnan Kastrati

**Affiliations:** 1 Deutsches Herzzentrum München, Technical University of Munich, Lazarettstrasse 36, 80636 Munich, Germany; 2 Institut Für Laboratoriumsmedizin, Deutsches Herzzentrum München, Technical University of Munich, Munich, Germany; 3 Medizinische Klinik Und Poliklinik Innere Medizin I, Klinikum Rechts Der Isar, Technical University of Munich, Munich, Germany; 4 German Center for Cardiovascular Research (DZHK), Partner Site Munich Heart Alliance, Munich, Germany

Sirs:

The influence of body size on platelet response to ticagrelor and prasugrel remains poorly investigated. So far, limited evidence exists on the platelet response to prasugrel in relation to body size indices [1]. In regard to ticagrelor, the platelet response to this drug has been investigated only according to body mass index (BMI) and the data are controversial [2–4]. We undertook this study to assess the influence of body size indices on the platelet response to ticagrelor and prasugrel in patients with acute coronary syndromes (ACS).

This study included 598 patients with ACS who were platelet P2Y_12_ receptor inhibitor-naïve on admission and who underwent platelet function tests in the setting of Intracoronary Stenting and Antithrombotic Regimen: Rapid Early Action for Coronary Treatment (ISAR-REACT) 5 trial (Clinical Trial Registration: NCT01944800) at the Klinikum rechts der Isar and Deutsches Herzzentrum München, Germany. Patients with ACS planned to undergo an invasive treatment strategy were randomized to ticagrelor (loading dose of 180 mg) or prasugrel (loading dose of 60 mg) with 1:1 randomization ratio. Detailed inclusion/exclusion criteria are reported in the primary publication [5]. Venous whole blood was obtained from patients before and after study drug loading dose administration. Adenosine diphosphate (ADP)-induced platelet aggregation values were measured using the Mulitplate^®^Analyzer (Roche Diagnostics, Switzerland). Platelet aggregation values were quantified as area under the curve of aggregation units (AU × min) [6].

Five body size indices were included in the current analysis: body weight (BW) in kg; BMI calculated as weight(kg)/height(m)^2^; body surface area (BSA) calculated as BSA = (weight[kg]^0.425^ × height[cm]^0.725^) × 0.007184 [7]; lean body mass (LBM) calculated as, LBM = (1.1 × weight[kg]) − 128 × (weight[kg]/height[cm])^2^ in men and LBM = (1.07 × weight [kg]) − 148 × (weight[kg]/height[cm])^2^ in women [8]; and blood volume (BV) calculated as, BV = (0.006012 × height[inch]^3^) /(14.6 × weight [pound] + 604 in men and BV = (0.005835 × height[inch]^3^) / (15 × weight [pound]) + 183 in women [9].

The primary outcome was ADP-induced platelet aggregation values within 24 h following loading dose of ticagrelor and prasugrel. Patients were categorized in subgroups according to tertiles of each body size index. Data are presented as medians with 25th–75th percentiles, mean ± standard deviation or counts (%). Comparison between groups was performed using the Kruskal–Wallis rank-sum test. A two-sided *P* < 0.05 was considered to indicate statistical significance. The study was approved by the Local Ethics Committee and conforms to the Declaration of Helsinki.

Baseline data are shown in Table 1. Baseline demographic, clinical and procedural characteristics were well balanced between patients in ticagrelor and prasugrel groups. All patients underwent percutaneous coronary intervention. Measurement of ADP-induced platelet aggregation was performed after a median [25th–75th percentiles] of 11.8 [5.4–16.9] hours after prasugrel loading and 11.8 [6.1–17.8] hours after ticagrelor loading (*P* = 0.299). ADP-induced platelet aggregation values at baseline and within 24 h after drug loading are shown in Table 2. Baseline ADP-induced platelet aggregation values did not differ significantly according to the tertiles of all body size indices in ticagrelor or prasugrel groups. A strong platelet inhibition was observed in ticagrelor and prasugrel groups and the platelet inhibition was stronger with prasugrel than ticagrelor. The deeper platelet inhibition with prasugrel versus ticagrelor in all tertiles of body size indices supports the main findings of the primary trial [5]. In the ticagrelor-treated patients, the ADP-induced platelet aggregation values within 24 h following drug loading did not to differ according to tertiles of each body size index (*P* ≥ 0.488 for all comparisons). In the prasugrel-treated patients, the ADP-induced platelet aggregation values were higher in upper tertiles of BW, BSA, LBM and BV, but not BMI. The level of statistical significance was achieved for BSA (*P* = 0.016), LBM (*P* = 0.042) and BV (*P* = 0.017; Table 2).

**Table 1:** Baseline characteristics

Characteristic (*n* = 598)	Value
Age (years)	65.0 [55.0–75.0]
Women	126 (21.1)
Diabetes mellitus	129 (21.6)
Current smoking	198 (33.7)
History of arterial hypertension	431 (72.6)
History of hypercholesterolemia	385 (64.4)
Previous myocardial infarction	109 (18.3)
Previous percutaneous coronary intervention	149 (25.0)
Previous coronary artery bypass surgery	40 (6.7)
Systolic blood pressure (mmHg)	150 [130–163]
Diastolic blood pressure (mmHg)	80 [75–90]
Unstable angina	64 (10.7)
Non-ST-segment elevation myocardial infarction	254 (42.5)
ST-segment elevation myocardial infarction	280 (46.8)
Coronary angiography	598 (100.0)
Femoral artery access	581 (97.2)
Radial artery access	17 (2.8)
Extent of coronary artery disease	
One-vessel disease	145 (24.2)
Two-vessel disease	172 (28.8)
Three vessel disease	281 (47.0)
Left ventricular ejection fraction (%)	48.7 ± 10.1
Post-procedural TIMI flow grade	
0	9 (1.5)
1	1 (0.2)
2	25 (4.2)
3	563 (94.1)
Percutaneous coronary intervention	598 (100.0)
Drug-eluting stent	500 (83.6)
Bioresorbable vascular scaffold	67 (11.2)
Periprocedural antithrombotic therapy	
Aspirin	559 (93.4)
Unfractionated heparin	596 (99.7)
Low molecular weight heparin	5 (0.8)
Bivalirudin	1 (0.2)
Beta-blocking agents on admission	180 (30.1)
Statins on admission	182 (30.4)
Body weight (kg)	81.0 [72.0–93.0]
Body mass index (kg/m^2^)	26.9 [24.7–29.7]
Body surface area (m^2^)	1.97 [1.83–2.10]
Lean body mass (kg)	58.9 [53.2–63.9]
Blood volume (Liter)	5.18 [4.62–5.64]

Data are median with 25th–75th percentiles, mean ± standard deviation or count (%)

*TIMI* thrombolysis in myocardial infarction

**Table 2:** Platelet aggregation measurement at baseline and within 24 h after drug loading dose administration

Body size index/ADP-induced platelet aggregation values	Ticagrelor (*n* = 289)			*P* value	Prasugrel (*n* = 309)			*P* value
BW tertiles	1 (*n* = 98)	2 (*n* = 95)	3 (*n* = 96)		1 (*n* = 109)	2 (*n* = 109)	3 (*n* = 91)	
BW (kg)	70.0 [62.2–73.0]	81.0 [80.0–85.0]	96.0 [93.0–106.0]	< 0.001	69.0 [63.0–72.0]	82.0 [80.0–85.0]	100.0 [95.0–108.0]	< 0.001
Baseline ADP-induced platelet aggregation values (AU × min)	862 [607–1112]	832 [593–1134]	798 [628–1122]	0.941	832 [555–1102]	833 [654–1124]	845 [632–1005]	0.751
After drug loading dose ADP-induced platelet aggregation values (AU × min)	130 [73.5–198]	147 [84.5–230]	126 [75–197]	0.488	88 [48–149]	116 [64–173]	117 [63–199]	0.054
BMI tertiles	1 (*n* = 97)	2 (*n* = 96)	3 (*n* = 96)		1 (*n* = 104)	2 (*n* = 102)	3 (*n* = 103)	
BMI (kg/m^2^)	23.8 [22.1–24.8]	26.8 [26.1–27.8]	31.1 [29.6–34.4]	< 0.001	23.7 [22.2–24.5]	27.1 [26.2–27.7]	31.4 [29.9–34.0]	< 0.001
Baseline ADP-induced platelet aggregation values (AU × min)	832 [608–1108]	867 [547–1144]	784 [634–1083]	0.927	833 [561–1105]	831 [638–1113]	843 [636–1018]	0.753
After drug loading dose ADP-induced platelet aggregation values (AU × min)	132 [77–212]	146 [86–221]	126 [68–195]	0.664	107 [58–169]	102 [59–180]	103 [60–160]	0.988
BSA tertiles	1 (*n* = 97)	2 (*n* = 96)	3 (*n* = 96)		1 (*n* = 103)	2 (*n* = 103)	3 (*n* = 103)	
BSA (m^2^)	1.77 [1.69–1.83]	1.97 [1.93–2.01]	2.16 [2.10–2.25]	< 0.001	1.75 [1.67–1.82]	1.97 [1.93–2.01]	2.15 [2.11–2.25]	< 0.001
Baseline ADP-induced platelet aggregation values (AU × min)	881 [607–1149]	786 [603–1098]	794 [625–1122]	0.554	848 [621–1105]	828 [590–1098]	836 [636–1008]	0.899
After drug loading dose ADP-induced platelet aggregation values (AU × min)	130 [86–198]	144 [80–224]	126 [75.5–202]	0.845	87 [47–140]	119 [66–174]	116 [63–199]	0.016
LBM tertiles	1 (*n* = 99)	2 (*n* = 94)	3 (*n* = 96)		1 (*n* = 103)	2 (*n* = 103)	3 (*n* = 103)	
LBM (kg)	51.8 [48.5–53.4]	58.9 [57.3–60.3]	66.1 [63.9–69.0]	< 0.001	50.0 [47.0–53.0]	59.0 [57.3–60.3]	66.0 [64.3–68.8]	< 0.001
Baseline ADP-induced platelet aggregation values (AU × min)	864 [596–1137]	798 [654–1115]	794 [600–1122]	0.838	857 [637–1118]	828 [569–1098]	822 [631–996]	0.508
After drug loading dose ADP-induced platelet aggregation values (AU × min)	129 [74–206]	144 [83–219]	135 [75.5–205]	0.764	88 [49–142]	119 [65–171]	117 [62.5–196]	0.042
BV tertiles	1 (*n* = 99)	2 (*n* = 96)	3 (*n* = 94)		1 (*n* = 103)	2 (*n* = 103)	3 (*n* = 103)	
BV (Liter)	4.42 [4.15–4.65]	5.20 [5.05–5.32]	5.86 [5.63–6.17]	< 0.001	4.25 [3.82–4.60]	5.18 [4.99–5.31]	5.82 [5.64–6.14]	< 0.001
Baseline ADP-induced platelet aggregation values (AU × min)	880 [607–1148]	784 [628–1095]	810 [611–1125]	0.551	858 [644–1148]	809 [543–1060]	824 [636–999]	0.209
After drug loading dose ADP-induced platelet aggregation values (AU × min)	130 [74–208]	134 [80–208]	142 [79–206]	0.858	88 [44–140]	119 [65.5–174]	117 [63–196]	0.017

Data are median with 25th–75th percentiles

*ADP* adenosine diphosphate, *BMI* body mass index, *BW* body weight, *BSA* body surface area, *BV* blood volume, *LBM* lean body mass

The main findings of this study may be summarized as follows: (1) Platelet response to ticagrelor appears not to depend on the body size. ADP-induced platelet aggregation values within 24 h following ticagrelor loading did not differ according to tertiles of body size indices investigated in the current study. (2) Platelet response to prasugrel appears to differ according to body size indices with higher platelet aggregation values in upper tertiles in four of five body size indices investigated in this analysis. (3) Platelet response to ticagrelor or prasugrel appears not to differ according to BMI tertiles, the commonly used body size index in pharmacological and clinical studies involving antiplatelet drugs. Thus, BMI may be suboptimal to guide dosing of antiplatelet drugs based on the platelet response to a given drug. Jakubowski et al.[1] reported an inverse relationship between body size indices (BW, BMI and BSA) and response to clopidogrel or prasugrel. Notably, the body size was a determinant of exposure to active metabolites and residual platelet activity regardless of type and dose of thienopyridine and BW and BSA showed stronger correlations with platelet reactivity [1]. With regard to platelet response to ticagrelor in relation to body size indices, a study by Deharo et al. [2] reported that BMI did not impact platelet inhibition by ticagrelor in patients with ACS. The frequency of drug administration (twice daily for ticagrelor and once daily for prasugrel) may impact on the platelet response to ticagrelor or prasugrel according to the body size. The study has limitations mostly related to being a subgroup analysis and to not repeating the platelet inhibition tests during the study course.

In conclusion, body size appears to influence the platelet response to prasugrel but not to ticagrelor. Among body size indices investigated, higher values of BSA, LBM and BV but not BMI were associated with higher ADP-induced platelet aggregation values within 24 h following prasugrel loading. These data may serve to optimize dosing of antiplatelet drugs in patients with ACS. Future studies are needed to confirm these results and clarify how antiplatelet drug dosing could be adjusted according to the body size.
